# Health status and living conditions of north-bound migrant minors in Mexico at place of origin and during the migration journey: A cross-sectional, mixed-methods study

**DOI:** 10.1016/j.jmh.2025.100390

**Published:** 2025-12-30

**Authors:** Zeus Aranda, Ana Cristina Sedas, Daniel Bernal, José Pulido-Manzanero, Enrique Regidor, Anna M. Mandalakas, Karla Fredricks

**Affiliations:** a Departamento de Salud Pública y Materno-Infantil, Facultad de Medicina, Universidad Complutense de Madrid, Calle Martín Lagos s/n, Madrid, 28040, España; b Universidad Autónoma de Piedras Negras, Padre de las Casas 83, Piedras Negras, 26000, México; c Division of Global Health, Department of Pediatrics, Baylor College of Medicine, 1102 Bates Avenue FC 630, Houston, TX, 77030, USA; d Center for Humanitarian Health, Johns Hopkins Bloomberg School of Public Health, 615 N. Wolfe Street, Baltimore, MD, 21205, USA; e Escuela de Gobierno y Transformación Pública, Instituto Tecnológico y de Estudios Superiores de Monterrey, *Av*. Revolución 756, Ciudad de México, 03700, México; f Department of Global Health and Development, London School of Hygiene & Tropical Medicine, Keppel Street, London, WC1E 7HT, UK; g CIBER de Epidemiología y Salud Pública (CIBERESP), *Av*. Monforte de Lemos 3-5, Madrid, 28029, España; h Instituto de Investigación Sanitaria del Hospital Clínico San Carlos, Calle del Prof. Martín Lagos s/n, Madrid, 28040, España

**Keywords:** Mexico, Latin America, Migrant health, Child health, Adolescent health

## Abstract

The passage of north-bound migrants through Mexico has increased dramatically in the last decade, with an increasing proportion of minors. However, there is still little evidence on the health status and living conditions of migrant minors in transit through Mexico. The aim of this study was to better characterize the evolution of health status and its contributing factors in this population from the place of origin to the period of travel. We conducted a cross-sectional mixed-methods study with migrant caregivers staying with minors in shelters in northern Mexico. Quantitative data were collected through surveys using validated tools to assess minors’ well-being and health-related social needs and were analyzed descriptively and inferentially. Qualitative data were obtained through focus groups using a semi-structured interview guide and analyzed through thematic analysis. Information was collected on 200 minors between July and September 2024. Caregivers reported precarious and unsafe living conditions at origin and along the journey, as well as a series of barriers to accessing health services. Deterioration in the mental and physical health of minors was observed between the pre-departure and travel periods. Our study illuminates adversities faced by migrant children and adolescents in Latin America along the migratory journey and emphasizes the impact of these adversities on their well-being. There remains a need to continue supporting the implementation of interventions that contribute to the well-being of minors in transit in the region.

## Highlights

• North-bound migrant minors in Mexico live in precarious and unsafe conditions in their place of origin that are perpetuated throughout the migratory journey.
• This population faces barriers to accessing health services at origin and during the journey.
• As a result of the adversities of travel, many experience deterioration in their physical and mental health.
• One of the main concerns of caregivers is the psycho-emotional well-being of their children.
• Caregivers suggested implementing specialized services in child health and activities that contribute to minors’ psycho-emotional well-being in migrant shelters.

## 1 Introduction

Human mobility in the Latin American and Caribbean region (LAC) has experienced a great increase in the first two decades of the twenty-first century. According to the United Nations Department of Economic and Social Affairs, the number of international migrants in the region increased from 24.6 million in 2000 to 42.9 million in 2020 (Population Division 2024). Migratory profiles are diverse, including asylum seekers, economic and environmental migrants, unaccompanied minors, and victims of human trafficking, many of whom are stranded in Mexico or other countries along the migration route due to border closures or asylum bans. (Esteinou, 2024) Migratory flows in the LAC over the last decade have been shaped by the forced migration of populations from South American countries such as Colombia and Venezuela, Central American countries such as Nicaragua and Honduras, and Caribbean countries such as Cuba and Haiti, because of natural disasters, political and economic conflicts, and violence exercised by the government, organized crime, or guerrillas (International Organization for Migration 2024; Selee et al., 2023; Aranda et al., 2025).

As the United States (US) remains one of the main destinations for emigrants from LAC countries, as well as for emigrants from other regions who journey to the US through the LAC, many countries in the region are transit corridors for these migratory flows. (International Organization for Migration 2024) This is the case of the Mexico-US corridor, the world’s largest migration route, with over 11 million migrants. (International Organization for Migration 2024) The growing number of migrants is reflected by the sharp rise in encounters between Mexican immigration authorities and people in an irregular migratory situation (who do not have an immigration document to prove their legal stay in the country), which increased from 66,583 in 2011 to 925,085 in 2024. (Gobierno de México 2025, Gobierno de México 2025) The increase in this population has been accompanied by a change in its demographic profile, with a notable increase in the percentage of people from South America (1.72 % in 2011 to 52.24 % in 2024) and the Caribbean islands (1.38 % in 2011 to 9.99 % in 2024), leading to a corresponding decrease in the percentage of people of Central American origin (93.4 % in 2011 to 27.9 % in 2024) (Gobierno de México 2025). Another notable change is the decrease in the proportion of males (86.24 % in 2011 to 68.2 % in 2024) (Gobierno de México 2025). as well as an increase in the proportion of minors—accompanied and unaccompanied combined—with respect to the total (6.25 % in 2011 to 14.58 % in 2023) (Gobierno de México 2024).

Migrant populations face tremendous adversity throughout their journey, negatively impacting their health. Detrimental effects are most likely to be significant in children and adolescents, who are more vulnerable to certain infectious diseases and adverse experiences due to their immature physical, immunological, and mental development. (Centers for Disease Control and Prevention 2022, Organisation for Economic Co-operation and Development 2019) The harsh conditions of travel (e.g., long journeys; lack of food, clean drinking water, and hygiene facilities; overcrowded conditions; and potential separation from caregivers) increase children and adolescents' exposure to infectious diseases and violence, which in turn increases their risk of health problems such as respiratory and gastrointestinal infections, injuries, as well as acute and chronic mental illness. (SAMHSA 2023) The vulnerability of children and adolescents is aggravated by their exclusion from national health promotion, disease prevention and care programs when they are in an irregular migratory situation. (UNICEF 2022)

There is a substantial evidence gap related to the health status and travel conditions of migrant minors transiting through the LAC. There are several studies describing the sociodemographic characteristics, travel conditions, health needs, and access to and use of health services of the migrant population in Mexico (El Colegio de la Frontera Norte 2022, The Migrante Project 2013, Martinez-Donate et al., 2020, Leyva Flores et al., 2016, López Arellano, 2014, International Rescue Committee 2019). However, in nearly all cases, the information is insufficient, focused on the adult population, and obsolete. The most recent and exhaustive source of information is the report of the United Nations International Organization for Migration (IOM), (OIM 2025) which only covers the issues superficially and does not have specific data on minors. Similarly, there is a significant lack of information from the country of origin describing the living conditions and health status of migrant minors in transit through Mexico; this baseline status greatly influences the migrants’ resilience and well-being during the journey and their final destination. (IOM 2023)

The lack of evidence of migrant children and adolescent health is not limited to the Mexican context. In 2023, the World Health Organization's (WHO) made a global call to action to address global evidence gaps for contextualized research on health and migration. The "Global Research Agenda on Health, Migration and Displacement "(WHO 2023) emphasized the importance of developing high-quality and policy-relevant research that would generate evidence on inclusive universal health coverage for migrants, address the determinants of migrants' health, and collect data on under-researched groups of migrants and their contexts, such as migrant children and adolescents. In complement, there have also been specific calls for information regarding migrant children and adolescents by other international agencies and local organizations in Mexico (Aranda and Mata, 2023). the US (Shastri, 2023), and other countries in the LAC (The Lancet Regional Health - Americas Editorial 2021, UNICEF 2023, Buechner, 2023, Marcus et al., 2023). In response to this dearth of information, this study aims to shed light on the current health status and health-related social needs of north-bound migrant minors in Mexico, evaluating both the situation in their place of origin and during their journey.

## 2 Methods

### 2.1 Study design

To broadly reflect the state of health and its determinants in the minor migrant population, we used a cross-sectional study design, collecting information on the period prior to the start of the journey (in the place of origin) and the period of travel in a single collection time (on Mexico's northern border) employing a convergent mixed methods design. The study and present manuscript were carried out according to the guidelines for STrengthening the Reporting of OBservational studies in Epidemiology (STROBE). (STROBE 2025)

### 2.2 Setting and participants

**Fig. 1:**
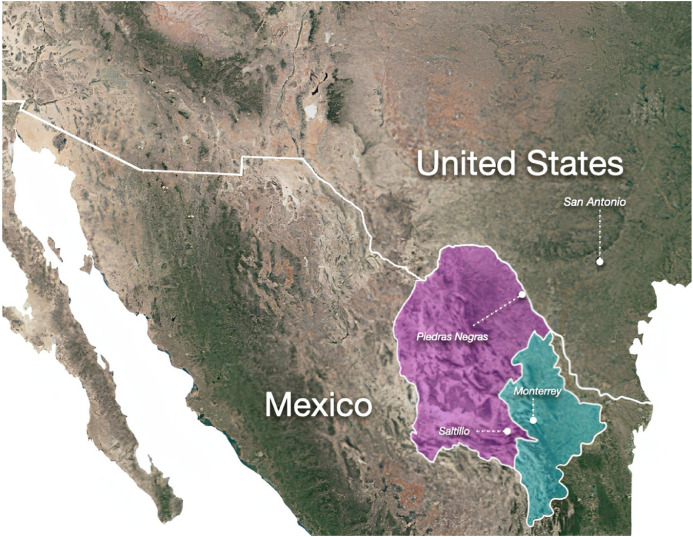
Map with the cities included in the study. In pink: Coahuila state. In blue: Nuevo León state.

The research was carried out in three major crossing areas within Mexico: the city of Piedras Negras in Coahuila, bordering the Texan city of Eagle Pass (US), as well as in Saltillo and the metropolitan area of Monterrey, capitals of Coahuila and Nuevo León states, respectively (Fig. 1). The three cities are part of the main migration routes by road in Mexico, as well as the rail network of freight trains used by part of the north-bound migrant population (Li Ng, 2020).

We indirectly gathered quantitative data on the health status and living conditions of migrant minors by administering detailed surveys to the primary caregivers accompanying them in shelters across the study sites. A unique survey was completed by the caregiver for each minor traveling with them. Limited access to the study population and its variable flow hindered the use of probability sampling methods, as reported in previous studies, therefore we opted for non-probabilistic sampling for convenience (Sulaiman-Hill and Thompson, 2011, Tyldum, 2021, Janse van Rensburg et al., 2021). Surveys were conducted to obtain information on at least 200 minors in transit to the US, aligning with the sample size used in IOM's Displacement Tracking Matrix (DTM) (OIM 2025) surveys in Mexico and similar studies with migrant populations in transit that have employed non-probability sampling methods.

For the qualitative component of the study, focus groups were conducted with caregivers staying in two shelters of Piedras Negras who had previously completed the survey. A convenience sample was employed. Focus groups were conducted until thematic saturation was reached.

### 2.3 Data collection

The survey questions were adapted from instruments previously used with populations similar to our participants. To assess the health status of minors, we used the short Spanish versions of the Child Health Questionnaire (CHQ) for children and adolescents aged 5 through 17 years and the Infant Toddler Quality of Life Questionnaire (ITQOL) for children aged 12 months to 5 years (see Supplementary Material for additional information). (QualityMetric 2024) The CHQ version consisted of 28 questions, while the ITQOL version consisted of 47 questions.

To characterize the health-related social needs of the minors within their country of origin, we used the Spanish proxy version of the "Accountable Health Communities Health-Related Social Needs Screening Tool" (AHC HRSN Screening Tool) (see Supplementary Material for additional information). (Centers for Medicare, and Medicaid Services 2023) The ten questions corresponding to the five core domains of the tool (living situation, food, transportation, utilities, and safety) were asked.

In addition to the aforementioned validated tools, the survey collected information on additional variables supporting further exploration of the health status and health-related social needs of migrant minors. Previous studies were used as a reference for the design of these questions (Leyva Flores et al., 2016, López Arellano, 2014, International Rescue Committee 2019, The Migrante Project 2013, El Colegio de la Frontera Norte 2022, Centro de Estudios Públicos 2023, Secretaría de Salud de México 2022, World Health Organization, and King’s College London 2011).

The content of the questionnaire used in the surveys was reviewed before its implementation by a group of health professionals external to the study, enhancing its feasibility and content validity. The questionnaire was in Spanish (the lead author’s first language and the language common among all participants) and read to participants by the lead author. Participants' responses were recorded in a CommCare-based mobile application developed by the first author.

Focus groups were facilitated by the lead author in Spanish, using a semi-structured focus group guide with topics including the health status of the minors, their access to and use of health services, the minors’ social context within their country of origin and transit, and caregiver suggestions for improving the well-being of minors in shelters. Each focus group discussion was audio recorded with prior verbal informed consent obtained.

The research was conducted in accordance with the principles of the Declaration of Helsinki. Ethical approval was obtained through the Research Ethics Committee of the School of Medicine of the Monterrey Institute of Technology and Higher Education (Mexico) and the Institutional Review Board of Baylor College of Medicine (US). All participants gave verbal informed consent before participating in the study.

### 2.4 Data analysis

A descriptive analysis was performed, reporting frequency and percentage for categorical variables and average and standard deviation for continuous variables. To obtain the score of the CHQ and ITQOL subscales (see Supplementary Table 1 for additional information on the subscales), we used the software provided by the company that owns these tools (PRO CoRE [QualityMetric, 2025]). Student's *t*-tests were performed to compare the value of the different CHQ and ITQOL subscales before and during the migratory journey. Tests in which a *p* value of <0.05 was obtained were considered statistically significant. To determine whether an individual had a certain social need with the AHC HRSN Screening Tool, the corresponding guide issued by the tool's developers was used. (Centers for Medicare, and Medicaid Services 2023) Except for the calculations performed with PRO CoRE, data cleansing and analysis were carried out in STATABE 18 (StataCorp, 2023).

For the qualitative component, the transcription of each recording was made with the support of Sonix software (Sonix Inc., 2025) and the analysis was carried out with the support of Dedoose v9.2.22 software (SocioCultural Research Consultants, 2025). For the analysis, we used the methodology of thematic analysis as described by Braun and Clarke. (Braun and Clarke, 2013) A deductive and inductive approach was used to identify themes, with themes emerging from the topics asked to the participants as well as other subjects that arose spontaneously in the focus groups.

Finally, following a parallel convergent mixed-methods research design, qualitative and quantitative findings were combined, compared, and interpreted together.

### 2.5 Role of funding source

No funding was received for this study.

## 3 Results

### 3.1 Sociodemographic characteristics of primary caregivers: quantitative component

The surveys were answered by a total of 118 primary caregivers of north-bound migrant minors (Table 1). Participants reported a mean age of 31.9 years and cared for an average of 1.69 minors each. Caregivers were mostly women (93.22 %), did not consider themselves indigenous (95.76 %) or Afro-descendant (94.92 %), and had completed secondary or higher education (58.47 %). Most caregivers had a support network at their destination in the US (88.98 %), with the main destination states being Texas (42.37 %), New York (8.47 %), and California (5.08 %).

**Table 1:** Sociodemographic characteristics of the primary caregivers participating in the surveys.

	Mean (SD)
	*n* = 118
**Age**	31.9 (7.37)
**Accompanying minors per caregiver**	1.69 (0.84)
	**n (%)**
**Gender**	
Male	8 (6.78)
Female	110 (93.22)
**Identifies as a member of an indigenous community**	
No	113 (95.76)
Yes	5 (4.24)
**Identifies as Afro-descendant**	
No	112 (94.92)
Yes	6 (5.08)
**First language**	
Spanish	117 (99.15)
Other	1 (0.85)
**Practicing any religion**	
No	24 (20.34)
Yes	94 (79.66)
**Highest level of education completed**	
None	2 (1.69)
Primary	47 (39.83)
Secondary	31 (26.27)
High school	23 (19.49)
University	15 (12.71)
**United States destination state**	
Texas	50 (42.37)
New York	10 (8.47)
California	6 (5.08)
Other	52 (44.08)
**Support network at destination in the US**	
No	13 (11.02)
Yes	105 (88.98)

### 3.2 Sociodemographic characteristics of primary caregivers: qualitative component

The four focus groups included 16 caregivers, self-identified as biological mothers, who were residing in shelters in Piedras Negras (Table 2). Participants had an average age of 32.5 years and primarily originated in Honduras (43.8 %), Guatemala (18.8 %), and Venezuela (12.5 %). The caregivers had an average of two minors in their care, with an average age of nine years amongst all minors.

**Table 2:** Sociodemographic characteristics of the primary caregivers participating in the focus group discussions.

	Mean (SD)
	*n* = 16
**Age of caregivers**	32.38 (6.66)
**Number of minors per caregiver**	2 (0.73)
**Age of minors**	8.95 (4.77)*
	**n (%)**
**Focus group**	
Group 1 shelter 2 Piedras Negras	5 (31.25)
Group 2 shelter 2 Piedras Negras	4 (25)
Group 3 shelter 1 Piedras Negras	4 (25)
Group 4 shelter 1 Piedras Negras	3 (18.75)
**Country of origin**	
Honduras	7 (43.75)
Guatemala	3 (18.75)
Venezuela	2 (12.5)
Colombia	1 (6.25)
Ecuador	1 (6.25)
México	1 (6.25)
Perú	1 (6.25)

*n = 32 minors.

### 3.3 Sociodemographic characteristics of minors and living conditions at origin

Caregivers who responded to the survey provided information on 200 minors (Table 3). Most of them were staying in shelters in Piedras Negras (65.5 %), followed by the metropolitan area of Monterrey (30.5 %), and Saltillo (4 %). In most cases, it was the mothers who responded to the survey (90 %). Four minors had been born on the journey (in Mexico) and the rest came mainly from Honduras (64.29 %), Venezuela (13.27 %), and Mexico (4.59 %). The mean age of the children was 7.41 years, and there was an approximately even split between male and female (52.5 % vs. 47.5 %, respectively).

**Table 3:** Sociodemographic characteristics of the minors and living conditions at origin.

	Mean (SD)
	*n* = 200
**Age**	7.41 (4.42)
	**n (%)**
**Born on the journey**	
No	196 (98)
Yes	4 (2)
**Gender**	
Male	105 (52.5)
Female	95 (47.5)
**Location of the shelter where the minor was staying**	
Piedras Negras	131 (65.5)
Metroplitan area of Monterrey	61 (30.5)
Saltillo	8 (4)
**Relationship of primary caregiver surveyed to the minor**	
Mom	180 (90)
Dad	12 (6)
Grandmother	7 (3.5)
Uncle	1 (0.5)
**Schooling before departure**	
No (not of compulsory school age)	65 (32.5)
No (with compulsory school age)	6 (3)
Yes	129 (64.5)
**Primary caregiver's reason for leaving (multiple choice)**	
Violence/insecurity	187 (93.5)
Economic problems	123 (61.5)
Education of minors	96 (48)
Family reunification	78 (39)
Political	62 (31)
Hunger/malnutrition	60 (30)
**Country of origin**	*n* = 196*
Honduras	126 (64.29)
Venezuela	26 (13.27)
Mexico	9 (4.59)
Colombia	8 (4.08)
Ecuador	8 (4.08)
Guatemala	8 (4.08)
Salvador	8 (4.08)
Peru	3 (1.53)
HRSN related to housing stability and/or housing quality at origin**	
No	68 (34.69)
Yes	128 (65.31)
**HRSN related to purchasing food at origin**	
No	46 (23.47)
Yes	150 (76.53)
**HSRN related to accessing reliable transportation at origin**	
No	111 (56.63)
Yes	85 (43.37)
**HRSN related to difficulty paying utility bills at origin**	
No	20 (10.2)
Yes	176 (89.8)
**HRSN related to child abuse at origin**	
No	122 (41)
Yes	78 (39)

HRSN: Health-Related Social Needs according to the "The Accountable Health Communities Health-Related Social Needs Screening Tool".

*Minors born during the journey excluded.

**Dwellings without smoke detectors were not considered to have an HRSN related to housing quality, since 98.98 % of participants did not have one.

Most minors of compulsory school age according to their countries of origin were in school before leaving (95.56 %). According to the focus groups, despite a high level of schooling among caregivers, most reported a poor education system for their children in their countries of origin. They mentioned teacher absenteeism, inaction in cases of bullying, or the application of disproportionate punishments as some of the main problems in public schools (Table 4, excerpt 1).

**Table 4:** Excerpts from the focus groups.

Results section	No. of excerpt	Excerpts
Sociodemographic characteristics of minors and living conditions at origin	1	*"They come home and tell me 'Mom, look that at school they bullied me I went to the teacher and told him', and no, they don't do anything. That's why children don't want to go to school because… look, my son is dark-skinned: 'Look, mommy, they told me I was like that', he didn't want to go to school."* (Group 1 shelter 2 Piedras Negras, Honduras)
	2	*"Where I lived was not safe, one had to be on the lookout, because as in the city it is not the same as in the village. The gangs bother you a lot, there are kidnappings, you have to be aware. You know, be with your windows closed and be on the lookout."* (Group 4 shelter 1 Piedras Negras, Venezuela)
	3	*"I had a belly like this* [represents being pregnant] *with the twins. And I was going to stand in line. A line like in Cuba.* […] *I dawned in the queues to buy* [food and personal hygiene products]*."* (Group 3 shelter 1 Piedras Negras, Venezuela)
Diagnosed health conditions and access to health services pre-departure	4	*"In my case my daughter is hyperactive. I paid part of it* [the health care] *and another part helped me in some foundations. So, it's like half paid and half that the foundation helps her. In Honduras, if you don't have money, you don't go to the psychologist."* (Group 1 shelter 2 Piedras Negras, Honduras)
Travel conditions	5	*"I asked for food, I wasn't embarrassed.* […] *I brought money, but it was to save, because I didn't know what was going to happen to me on that road."* (Group 3 shelter 1 Piedras Negras, Venezuela)
	6	*"Today they didn’t give breakfast* [to adults]*.* […] *And the little children are on a glass of coffee until four* [from nine]. (Group 3 shelter 1 Piedras Negras, Venezuela)
	7	*"There the three of us got sick with diarrhea. Eh, well, I say it was the water because when I first arrived, I drank a lot of that water that was supposedly filtered.* […] *And the next day we all woke up with diarrhea."* (Group 4 shelter 1 Piedras Negras, Venezuela)
	8	*"Here* [in one of the shelters] *I don't feel safe. You don't know if suddenly the demons come and jump that wall. We are here as if we are out in the open."* (Group 3 shelter 1 Piedras Negras, Venezuela)
	9	*"After passing that ranch, there was a man with a machete, and then you have to pay or go back.* […] *The cartel has taken money from me, of course, because you don't want to get killed."* (Group 4 shelter 1 Piedras Negras, Venezuela)
	10	*"We were living in Monterrey; we were there for a long time. We went to some barracks and the one who lived there was Mexican and she bothered us verbally, exaggerated, and all that was missing was for her to hit us. And she said, 'These catrachos* [colloquial demonym synonymous with Honduran] *that don't leave me, that I don't know what they're doing here.' Total discrimination. And nobody, nobody did anything to her."* (Group 1 shelter 2 Piedras Negras, Honduras)
	11	*"When migration chased us, my baby went white with fright. And until I pulled him, because if not he was going to stay there.* […] *Running, we left. My brother helped me carry the girl."* (Group 3 shelter 1 Piedras Negras, Guatemala)
Health discomfort, diagnosis of health conditions and access to health services on the journey	12	*"Yes, it has affected them a lot, because… An example, the children did stop eating, they were just thinking about the same thing* [traumatic situation that the participant preferred not to specify]*, the same thing and always with that fear that the same thing could happen to them."* (Group 1 shelter 2 Piedras Negras, Guatemala)
	13	*"In our country there was more freedom. They went to school, here they haven’t gone to school, and they haven’t had much freedom. So, there you meet more people, more friends, all that. But since they have come here, one changes, because it is no longer our country. Things are different. They deprive themselves of what they are used to doing day by day."* (Group 1 shelter 2 Piedras Negras, Honduras)
	14	*"My girl, she tells me 'Mom, I want us to be in one place. I don't want us to walk around like this anymore.' It's a trauma for her." (Group 1 shelter 2 Piedras Negras, Honduras)*
Utilization of health services during the journey	15	*"I can't complain about those doctors. They gave me gauze, they gave me everything to cure her, they gave her an injection. Everything. I didn't pay anything."* (Group 3 shelter 1 Piedras Negras, Venezuela)
Proposals for improving the well-being of migrant minors in shelters	16	*"I gave in to them having a phone, but yes, I mean, it doesn't bring them benefits, but it's a way to keep them entertained for us here in this place, because we don't see how else to entertain them.* […] *If, for example, there was someone professional who came to give them some classes, then they are going to say 'Well, I have to go to the class and I go and that's it'."* (Group 1 shelter 2 Piedras Negras, Guatemala)
	17	*"For the children who have had to go through this journey that we have gone through, I think that they should be attended by a psychologist.* […] *Well, being one who has gone to the psychologist, you still can't get that out of your mind. Not to mention the children who have not gone in to the psychologist. They still have that working in their minds.* […] *So, for me, I believe that children also have to be treated with a psychologist so that they can express what they feel.* […] *The problem is that sometimes the child is silent about what he thinks, what he feels. They don't express it; they keep quiet about it."* (Group 1 shelter 2 Piedras Negras, Honduras)
	18	*"I would ask for a constant doctor. A constant doctor for the children at least or twice a week who come here, to any shelter that can give help and bring them their vitamin. Because many children no longer take vitamins, many children need that, calcium, things like that."* (Group 3 shelter 1 Piedras Negras, Venezuela)

According to the surveys, the primary caregivers’ main reasons for leaving the place of origin were violence or insecurity (93.5 %), economic problems (61.5 %) and the search for better education for the minors in their care (48 %). Insecurity as one of the main reasons for migrating coincides with what was described in the focus groups, where several participants mentioned insecurity in their communities of origin and a climate of violence, both due to the presence of organized crime and the persecution of political dissidents by the authorities (Table 4, excerpt 2).

A large portion of the minors had health-related social needs in terms of unstable or poor-quality housing (65.31 %), food instability (76.53 %), unreliable transportation (43.37 %), difficulty paying utility bills (89.8 %), and abuse (39 %) in their place of origin (Table 4 and Supplementary Table 2). Material shortages were also expressed in the focus groups, where most of the caregivers agreed on the lack of access to food, clothing, personal hygiene items, and school supplies in their place of origin, especially the participants from Honduras and Venezuela, due to shortages and lack of purchasing power. Some participants reported the need to queue for hours to obtain basic products (Table 4, excerpt 3).

### 3.4 Diagnosed health conditions and access to health services pre-departure

Before leaving, 3 % of the minors had been diagnosed with a disability (neurodevelopmental disorders being the most common), with 66.67 % receiving the recommended care; 17 % with a non-communicable disease (asthma being the most common), with 55.88 % receiving the recommended care; and 2.5 % with an infectious disease in the year before departure (with stomach infections being the most common), with 100 % receiving the recommended care (Table 5 and Supplementary Table 3).

**Table 5:** Diagnosed health conditions and access to health services.

	Origin	Journey
	n ( %)	n ( %)
**Disability diagnosed by health professional**	*n* = 200	
No	194 (97)	200 (100)
Yes	6 (3)	0
Received appropriate care (recommended follow-up by health professional and treatment)	*n* = 6	*n* = 2*
No	2 (33.33)	2 (100)
Yes	4 (66.67)	0
**Non-infectious disease diagnosed by health care professional**	*n* = 200	
No	166 (83)	196 (98)
Yes	34 (17)	4 (2)
Received care (recommended follow-up by health professional and treatment)	*n* = 34	*n* = 25*
No	15 (44.12)	6 (24)
Yes	19 (55.88)	19 (76)
**Infectious disease diagnosed by health care professional**+	*n* = 200	
No	195 (97.5)	181 (90.5)
Yes	5 (2.5)	19 (9.5)
Received care (recommended follow-up by health professional and treatment)	*n* = 5	*n* = 20*
No	0	4 (20)
Yes	5 (100)	16 (80)
**Complications of disabilities and illnesses diagnosed prior to travel**	··	*n* = 45
No	··	40 (97.5)
Yes	··	5 (2.5)
**All vaccinations as recommended by country of origin at the time of the survey**	··	*n* = 200
No	··	12 (6)
Yes	··	188 (94)
**Always received the care he/she needed if he/she had a health need**+	*n* = 193**	*n* = 170**
No	42 (21.76)	28 (16.47)
Yes	151 (78.24)	142 (83.53)
Reasons for not receiving care (multiple choice)	*n* = 42	*n* = 28
Treatment not available	25 (59.52)	2 (7.14)
Could not afford it	23 (54.76)	9 (32.14)
Specialist not available	12 (28.57)	2 (7.14)
Was far away	8 (19.05)	3 (10.71)
Did not have health insurance	5 (11.9)	3 (10.71)
Did not know where to go	1 (2.38)	22 (78.57)
Could not use health facilities where treatment was available	··	1 (3.57)
Afraid of being reported to authorities and deported	··	1 (3.57)
Not allowed to leave the shelter	··	1 (3.57)
Not given a prescription for medicine	··	1 (3.57)

*Conditions diagnosed before and during the journey. Individuals without consultations and treatment needed for their health conditions were excluded.

**Individuals without health care needs excluded.

+ At origin, it only refers to the year prior to departure.

Approximately three-quarters of the minors had received the necessary care when they had any health needs in the year prior to departure (78.24 %). In case of having needs and not receiving care, the main causes were the lack of availability of treatment (59.52 %), not being able to afford it financially (54.76 %), and the lack of health professionals in their area (28.57 %).

In the focus groups, all participants agreed that public health care was poor in their home countries. Among the main problems they mentioned were long waiting times, lack of supplies, or poorly trained health personnel. Some of the caregivers had used the private health system, either through health insurance coverage, out-of-pocket costs, or with the support of civil society organizations (Table 4, excerpt 4).

### 3.5 Travel conditions

The average travel time of the minors was 6.85 months at the time of the survey, with 6.27 months in Mexican territory. The main form of travel was by motorized land transport (73.5 %), followed by foot (15 %), and train (11.5 %). Twenty-one percent of the minors had crossed the Darien Gap, approximately 100 km of jungle between Colombia and Panama that must be traversed on foot and is known to be especially dangerous (Table 6 and Supplementary figure 1). (Naranjo et al., 2023)

**Table 6:** Travel conditions.

	Mean (SD)
	*n* = 200
**Total travel time since child's departure (months)**	6.85 (3.73)
**Child's travel time in Mexico (months)**	6.27 (3.91)
	**n (%)**
**Main form of travel**	
Motorized land transport (truck, bus, car, etc.)	147 (73.5)
On foot	30 (15)
Train	23 (11.5)
**Crossed the Darien Gap**	
No	158 (79)
Yes	42 (21)
**Economic situation of primary caregiver at the time of the survey**	
Very poor/poor	146 (73)
Neither poor nor good	43 (21.5)
Good/very good	11 (5.5)
**Caregiver's sense that minor was safe/protected**	
Less than half of the days	151 (75.5)
Most days (half of the days or more often)	20 (10)
Every day	29 (14.5)
**Sleeping indoors**	
Less than half of the days	99 (49.5)
Most days (half of the days or more often)	61 (30.5)
Every day	40 (20)
**Access to toilets**	
Less than half of the days	100 (50)
Most days (half of the days or more often)	58 (29)
Every day	42 (21)
**Enough drinking water**	
Less than half of the days	90 (45)
Most days (half of the days or more often)	45 (22.5)
Every day	65 (32.5)
**Enough nutritious food (fruits, vegetables, tortillas, beans, unprocessed foods, etc.)**	
Less than half of the days	116 (58)
Most days (half of the days or more often)	39 (19.5)
Every day	45 (22.5)
**Access to hygiene measures/products (shower, soap, laundry, etc.)**	
Less than half of the days	141 (70.5)
Most days (half of the days or more often)	23 (11.5)
Every day	36 (18)
**Sufficient clothing, appropriate for the weather**	
Less than half of the days	127 (63.5)
Most days (half of the days or more often)	29 (14.5)
Every day	44 (22)

For most minors, the economic situation of their primary caregivers at the time of the survey was poor or very poor (73 %). The lack of resources led some caregivers to ask for money or food on public roads (Table 4, excerpt 5). Many of the minors experienced significant shortcomings during most of the travel time, including not sleeping indoors (49.5 %), not having access to bathrooms (50 %), not having enough water to drink (45 %), not having enough nutritious food (58 %), not having access to hygiene products/measures (70.5 %), and not having enough clothing (63.5 %). Similarly, all the participants in the focus groups mentioned having lived with material deprivation throughout the journey. Even in the shelters, shortages were common, with insufficient and dirty bedding, insufficient personal hygiene products, and scarce and non-nutritious food noted as concerns (Table 4, excerpt 6). Some caregivers reported sleeping outdoors at times, with extreme rain, heat, or cold. The lack of water suitable for human consumption was a recurrent problem noted—especially for those who had crossed the Darien Gap—which in some cases led to them and their families becoming ill (Table 4, excerpt 7).

According to survey data, most caregivers felt that minors were unsafe most of the travel time (75.5 %). This is consistent with what was expressed in the focus groups, where most participants reported feeling unsafe throughout the journey, even while staying in shelters (Table 4, excerpt 8). The participants reported having been exposed to different types of violence, both themselves and the minors in their care. Several participants mentioned having been victims of different criminal acts, including robbery, fraud, attempted kidnapping, and extortion by organized crime and the authorities (Table 4, excerpt 9). Some caregivers reported discriminatory treatment towards themselves and their children by other migrants (Table 4, excerpt 10). Several participants reported persecution by immigration authorities and police in Mexico, having to flee from them with their families to avoid being returned to a point closer to the border between Mexico and Guatemala (which would cause them to have to repeat the journey, accumulating fatigue and expenses) (Table 4, excerpt 11).

### 3.6 Health discomfort, diagnosis of health conditions and access to health services on the journey

During the journey, no child was diagnosed with a new disability, but four were diagnosed with a non-communicable disease (anemia being the most common), and 19 with an infectious disease (ear infections being the most common). Five (2.5 %) minors with a health condition diagnosed before departure experienced complications during the journey. None of the children with disabilities diagnosed before the journey received the recommended care during the journey, while 76 % of the cases of non-communicable diseases and 80 % of the cases of infectious diseases diagnosed before and during the journey received the recommended care while traveling.

Sixty-seven (33.5 %) minors had sustained some type of injury, although most had no need for medical attention. The most common injuries were burns, contusions, and lacerations (Table 7 and Supplementary figure 2). Most minors had a health concern during the journey (95.5 %), with the most prevalent physical conditions being cough, sore throat, and stomach pain and the most prevalent mental health conditions being sadness, nervousness or stress, and difficulty sleeping (Table 7 and Supplementary figure 3). This is in line with what was expressed in the focus groups, where most of the caregivers reported psychological and/or emotional distress of the minors due to the adversities faced throughout the migratory journey, manifesting itself in the form of low mood, behavioral changes (sleeping a lot and eating little), and repetitive negative thoughts (Table 4, excerpt 12).

**Table 7:** Injuries and health concerns during the journey.

	n ( %)
**Injured during the journey (lacerations/burns/fractures/sprains/contusions/blisters on feet)**	*n* = 200
No	133 (66.5)
Yes, no need for medical attention	56 (28)
Yes, with need for medical attention	11 (5.5)
**Any health concern during the journey**	
No	9 (4.5)
Yes	191 (95.5)
**Physical health concerns with highest prevalence**	
**Cough**	
Never	46 (23)
Some days (less than half the days)	153 (76.5)
Half the days or more often	1 (0.5)
**Sore throat**	
Never	63 (31.5)
Some days (less than half the days)	137 (68.5)
Half the days or more often	0
**Stomach pain**	
Never	83 (41.5)
Some days (less than half the days)	113 (56.5)
Half the days or more often	4 (2)
**Mental health concerns with highest prevalence**	
**Sadness**	
Never	91 (45.5)
Some days (less than half of the days)	48 (24)
Half the days or more often	61 (30.5)
**Nervousness or stress**	
Never	102 (51)
Some days (less than half the days)	33 (16.5)
Half the days or more often	65 (32.5)
**Insomnia/difficulty sleeping**	
Never	140 (70)
Some days (less than half the days)	26 (13)
Half the days or more often	34 (17)

Apart from exposure to different forms of violence, the minors’ mental well-being was also impacted by leaving their relatives, friends, activities, and material things in their country of origin and arriving in new places where they did not know anyone and were limited in the activities they could do (Table 4, excerpt 13). Furthermore, the situation of uncertainty was difficult, waiting for the resolution of migratory procedures and in continuous movement (Table 4, excerpt 14).

Regarding access to health services, most of the minors had received the necessary care when they had any health needs during the journey (83.53 %). In case of having needs and not receiving care, the main causes were not knowing where to go (78.57 %), not being able to afford it financially (32.14 %), far distance to the point of care (10.71 %), and lack of health insurance (10.71 %).

### 3.7 Utilization of health services during the journey

Most of the minors received at least one health consultation on the journey (79 %), with six children requiring hospitalization (Table 8). The average number of consultations per minor was 3.27. The main places of consultation were shelters (50.5 %), mobile clinics outside shelters (15.5 %), and public health centers (13 %). Most of the users of the services did not have to pay (87.97 %), did not perceive discrimination against minors by health personnel (99.37 %), and considered that the treatment of the providers had been good or very good (94.31 %). These findings are aligned with the positive experiences shared by several of the participants in the focus groups whose children received health care on the way (Table 4, excerpt 15).

**Table 8:** Health service utilization during the journey.

	Mean (SD)
	*n* = 200
**Number of health consultations**	3.27 (3.42)
	**n (%)**
**At least one health consultation**	
No	42 (21)
Yes	158 (79)
**Hospitalization**	
No	194 (97)
Yes	6 (3)
**Places of health consultation (multiple choice)**	
Shelter	101 (50.5)
Mobile clinic (health care tent)	31 (15.5)
Health center	26 (13)
Private clinic	19 (9.5)
Public hospital	15 (7.5)
Private hospital	1 (0.5)
Laboratory	1 (0.5)
Payment for health services	*n* = 158
No	139 (87.97)
Yes	19 (12.03)
Perceived discrimination from health care providers	
No	157 (99.37)
Yes	1 (0.63)
Treatment by health care providers	
Very poor	2 (1.27)
Poor	0
Average	7 (4.43)
Good	44 (27.85)
Very Good	105 (66.46)

### 3.8 Changes in the health status of minors between the place of origin and the migratory journey

Children aged 1–5 years, when comparing their health status during the journey with respect to the place of origin, were more limited in their physical activities due to health problems (decrease in the physical abilities scale from an average of 96.41 at origin to 93.03 on the journey; *p* = 0.0038), had more limiting pain (decrease in the body pain/discomfort scale from 94.61 to 71.81; *p* < 0.0001), had worse behavior (decrease in the global behavior scale from 73.73 to 60; *p* = 0.0008; and on the combined behavior scale from 80.33 to 71.42; *p* = 0.0001), worsened their health according to the caregiver's perception (decrease in the minor's global health perception scale from 75.42 to 70.6; *p* = 0.019), had increased caregiver concern about the child's health or development (decrease in the emotional scale of parental impact from 93.38 to 87.87; *p* = 0.0028), and the family's ability to get along worsened (decrease in the family cohesion scale from 75.1 to 66.57, *p* = 0.0279) (Table 9).

**Table 9:** Comparison of health status at origin vs. during the journey.

	Origin	Journey	
	n ( %)	n ( %)	
**General health status of children under 1 year old**^1^	*n* = 8		
Poor	1 (12.5)	1 (12.5)	
Fair	1 (12.5)	2 (25)	
Good	4 (50)	1 (12.5)	
Very good	0	1 (12.5)	
Excellent	2 (25)	3 (37.5)	
	**Mean (SD)**	**Mean (SD)**	***p*-value**
**Health-related quality of life of children aged 1 to 5 years old**^2^^,^** (score range)	*n* = 51		
Overall Health Scale (0–100)	57.94 (29.4)	58.92 (28.33)	0.8366
Physical Abilities Scale (0–100)	96.41 (7.52)	93.03 (10.88)	*0.0038
Growth and Development Scale (0–100)	91.57 (23.63)	95.2 (13.71)	0.0848
Bodily Pain/Discomfort Scale (0–100)	94.61 (17.55)	71.81 (28.16)	*<0.0001
Temperament and Moods Scale (0–100)	70.1 (13.94)	67.16 (14.57)	0.216
Global Behavior Scale (0–100)	73.73 (25.55)	60 (26.55)	*0.0008
Combined Behavior Scale (0–100)	80.33 (16.29)	71.42 (19.35)	*0.0001
Global Health Perception Scale (0–100)	75.42 (28.88)	70.6 (27.56)	*0.019
Parental Impact - Emotional Scale (0–100)	93.38 (12.95)	87.87 (19.66)	*0.0028
Parental Impact - Time Scale (0–100)	98.2 (4.81)	96.9 (6.66)	0.1176
Family Cohesion Scale (0–100)	75.1 (23.1)	66.57 (28.36)	*0.0279
**Health-related quality of life of children aged 5 to 17 years old**^2^^,^***	*n* = 141		
Global Health Scale (0–100)	62.55 (30.95))	60.78 (25.14)	0.5636
Physical Functioning Scale (0–100)	95.98 (13.17)	96.53 (14.31)	0.6482
Role/Social Limitations - Emotional Behavior Scale (0–100)	78.96 (34.14)	72.81 (35.55)	*0.0369
Role/Social Limitations - Physical Scale (0–100)	95.74 (14.28)	95.98 (15.67)	0.8765
Bodily Pain/Discomfort Scale (0–100)	95.46 (15)	81.13 (24.7)	*<0.0001
Behavior Scale (0–100)	78.33 (24.79)	75.33 (25.2)	0.0737
Global Behavior Scale (0–100)	68.65 (27.21)	63.72 (25.82)	*0.0348
Mental Health Scale (0–100)	72.34 (26.58)	71.22 (25.44)	0.5659
Self Esteem Scale (0–100)	88.47 (19.23)	54.49 (17.38)	*<0.0001
General Health Perception Scale (0–100)	79.29 (27.05)	82.26 (20.47)	0.1252
Parental Impact - Emotional Scale (0–100)	84.66 (22.82)	83.42 (23.07)	0.5615
Parental Impact - Time Scale (0–100)	97.04 (9.4)	96.22 (9.83)	0.3798
Family Activities Scale (0–100)	97.43 (11.33)	96.99 (11.27)	0.6537
Family Cohesion Scale (0–100)	71.24 (24.76)	66.35 (24.93)	*0.0075
Physical Component Summary Measure (-0.5–64)+	56.44 (9.91)	57.19 (9.81)	0.4074
Mental Component Summary Measure (6–64)+	50.95 (12.54)	44.93 (13.75)	*<0.0001

*p < 0.05.

**47 Item Short Infant and Toddler Quality of Life Questionnaire Parent Form (ITQOL-SF47).

***28 Item Short Child Health Questionnaire Parent Form (CHQ-PF28).

1 Origin=before the child’s departure or as a newborn (if born on journey); Journey=at the time of the survey.

2 Origin: within 4 weeks prior to departure; Journey=within 4 weeks prior to survey or during the entire journey if <4 weeks had elapsed since departure.

+ Norm-based scoring (NBS) is used to calculate the two summary scores. NBS standardizes component scores using the means and standard deviations from a United States (US) general population normative sample. The aggregated scores are standardized using a linear t-score transformation (mean of 50 and a standard deviation of 10). Summary scores below 50 can be interpreted as being below the US general population norm (average), while scores above 50 can be interpreted as above the US general population norm.

Comparing the travel period with the pre-departure period, minors aged 5 to 17 years had more behavioral or emotional problems (decrease in the role/social limitations scale of emotional behavior from an average of 78.96 at origin to 72.81 on the journey; *p* = 0.0369), had more limiting pain (decrease in the body pain/discomfort scale; *p* < 0.0001), had worse behavior (decrease in the global behavior scale from 68.65 to 63.72; *p* = 0.0348); had lower self-esteem (decrease in the self-esteem scale from 88.47 to 54.49; *p* < 0.0001), had a worsening of the family's ability to get along (decreased family cohesion scale from 71.24 to 66.35, *p* = 0.0075), and experienced overall mental decline (decreased mental component summary scale from 50.95 to 44.93; *p* < 0.0001) (Table 9).

### 3.9 Proposals for improving the well-being of migrant minors in shelters

A multitude of caregivers shared the need to implement enriching and educational initiatives aimed at minors in shelters, to keep them entertained with activities that bring them psychological and emotional benefits and to avoid educational lag. According to many caregivers, the lack of activities leads minors to spend many hours on the phone, which many considered to have a negative effect on their mental well-being (Table 4, excerpt 16). Several participants also mentioned the need for assistance from doctors and psychologists trained to provide specialized care to children and adolescents in shelters, as well as the delivery of nutritional supplements for minors in these spaces (Table 4, excerpts 17 and 18).

## 4 Discussion

Our study elucidates the precarious and unsafe conditions in which north-bound migrant minors from the LAC live in their places of origin and during the journey, as well as the deterioration of their physical and mental health along the migratory route.

After fleeing harmful living conditions in their countries of origin, the minors in our study experienced additional adverse events during the journey, including persecution, coercion, violence, and discrimination, as well as unsanitary living and transit conditions, food insecurity, and exposure to environmental hazards, leading to increased physical and psychological discomfort. The stages of the journey with the most negative experiences reported by the caregivers were the Darien Gap and the journey through Mexico, considered the most dangerous migratory routes in the Americas. (Hernández Ramia, 2024) Various organizations working on the ground have reported on the dangers faced by children and adolescents in these enclaves: In the Darien Gap, migrants often encounter criminal groups that rob or rape them, rushing rivers, and wild animals; (Naranjo et al., 2023) in Mexico, they are frequently subject to robbery, torture, extortion, sexual violence and exploitation, recruitment by organized crime, forms of child labor, and dangerous forms of transportation such as traveling on freight trains or in windowless truck containers. (Red por los Derechos de la Infancia en México REDIM 2025) There is serious risk for an even greater worsening in migrants’ health if conditions in countries of origin remain untenable and options for pathways to safe haven are limited further. Examples of concern include the closing of migratory support centers at the exit from the Darien Gap, increased measures to deter and deport migrants by immigration authorities in Mexico and the US, and the reduction in the right of asylum and humanitarian relief for individuals and families at risk of persecution or harm (Agencia EFE 2025, San Juan-Flores and Santos-Cid, 2025, MSF Colombia 2025).

The elimination of the CBP (US Customs and Border Protection) One system—which allowed asylum seekers to schedule appointments with US immigration authorities at various points along the southern US border to enter the country legally—on January 20, 2025, has had negative consequences for the health of northbound migrants, including children and adolescents. (Doctors Without Borders 2025) Apart from the impact of the program's closure on the mental health of migrants, this population is now forced to use unofficial, more dangerous routes to cross into the US, with a greater risk of injury and increased exposure to extortion, human trafficking, and other forms of violence. Similarly, for those stranded in high-risk areas of Mexico's northern border, their exposure to violence perpetrated by organized crime and authorities is prolonged, as are precarious living conditions and lack of access to basic services such as education and healthcare (Doctors Without Borders 2025).

The accumulation of adversities before and during the journey experienced by a large portion of the migrant children and adolescents in our study is reflected in the deterioration of their health status, especially their mental health, as has been observed in other studies with migrant children and adolescents from the LAC (Carreño Calderon et al., 2024, Andrade et al., 2023, Mixed Migration Centre 2023). The caregivers were concerned about the neglect of this problem in shelters, emphasizing the importance of implementing initiatives that promote the psychological and emotional well-being of minors. In select transit locations for the migrant population in Mexico and some other countries in the LAC, initiatives have been implemented for the psychosocial support of children and adolescents (Gobierno de México 2021, México, 2024, Guzmán-Carrillo et al., 2020, La Jugarreta 2025), although evidence on their impact is scarce (Marcus et al., 2023). Limitations of these initiatives include discontinuity—exacerbated by cuts in programs aimed at the migrant population in the Americas— (Agencia EFE 2025, Hernández, 2025). as well as their focus on health promotion and disease prevention without improving access to specialized child mental health care services during the migration journey (Marcus et al., 2023).

Despite the overall negative experiences in country of origin and throughout the migration journey, our study also showed that the participants had positive encounters when needing to access health services for their children. In contrast to the shortcomings of the health systems of the two main countries of origin of participants, Venezuela and Honduras (OECD/The World Bank 2020). migrants in our study were more likely to be able to access the care their children needed during the journey. Furthermore, in most cases, they were able to do so without cost or discrimination. This contrasts with existing evidence from LAC countries (primarily describing adults) where a significant proportion of migrant participants perceived discriminatory treatment in health services, mainly linked to their skin color or ethnic origin (Keys et al., 2019; Oyarte et al., 2023). The sociodemographic characteristics of participants in our study (e.g., Spanish-speaking, from the LAC) may be related to the low perceived discrimination in health services.

Our findings highlight the success of the civil society-led system of health care available to migrants through shelters and mobile clinics, suggesting that an expansion of services (to include mental health specifically and chronic disease care in general, especially neglected during the trip according to participants) might be effective in improving the health and well-being of migrants before they reach their final destination. These spaces also represent a good opportunity to identify complex, neglected health conditions that require specialized pediatric services, such as neurodevelopmental disorders. Due to the complexity of treating these conditions on site and considering the great importance of monitoring them, it would be important for shelters and mobile clinics to have a network of professionals to whom these cases can be referred.

Among participants who were not able to receive the necessary care for their children during the journey, the main barrier cited was not knowing where to access care. This barrier could be overcome through the implementation of information campaigns on the health services available along the migratory route for children and adolescents. Although campaigns of this type have already been carried out in Mexico and other LAC countries, their sporadic nature and the lack of specific information for each locality have limited their impact. (OPS/OMS 2022)

Considering the poor hygiene conditions, poor access to personal hygiene measures and products, and access to drinking water throughout the migration journey reported by participants, it would be important to promote WASH (Water, Sanitation, and Hygiene) programs in shelters and other spaces that house migrant families. This would promote decent living conditions and prevent health problems related to waterborne, foodborne, and vector-borne diseases (Scholz, 2016), such as those reported by multiple participants. On the other hand, given the limited access of the minors to nutritious and sufficient food throughout the migration journey, including in shelters, nutritional literacy sessions could be organized with minors and their caregivers, as well as with shelter staff when food services are available. This could help optimize the limited food resources available, maximizing nutritional benefits for the minors, as has been reported in previous studies with migrant populations in situations of nutritional precariousness (Seng et al., 2022).

### 4.1 Limitations

Due to the difficulty in fully characterizing and accessing the population in a situation of mobility in the context of the study, our study employed convenience sampling which may introduce bias. Further, the results observed may not be fully generalized to migrants outside of the shelters in the localities where the project was carried out. There are numerous migratory routes that may vary by the migrants’ origin and nationalities. For example, in the state of Baja California, a greater presence of migrants in an irregular migratory situation from the Caribbean Islands, Asia, and Africa has been identified than in Coahuila and Nuevo León. (Gobierno de México 2025) Hence, our findings may not be fully representative of other locations on Mexico’s northern border or other migrant populations. Finally, due to the high variability in the context of origin, journey, and political climate, the findings of the study may have limited representation of other time periods. Nevertheless, our robust methods and use of validated tools enhance the reliability, validity, and reproducibility of the data, thereby favoring generalizability to other populations of migrant children and adolescents.

Another limitation is the potential bias in the participation and responses of caregivers due to the state of vulnerability in which they found themselves. They may have felt conditioned to participate in the study or give certain answers to avoid conflicts with the organizations that provided them with help, to avoid being reported to immigration authorities, or to try to obtain preferential treatment. To minimize this bias, the collector emphasized that their participation would not benefit them directly, that the study was independent of the aid provider organizations, and that all information provided would be confidential and the data would be de-identified.

Finally, responses by caregivers rather than by the minors themselves, as well as the retrospective collection of pre-departure data, may have led to measurement and recall bias.

## 5 Conclusion

Our study demonstrates the adversities that migrant children and adolescents in the shelters experienced in different migratory stages as well as the detrimental effects of the migration journey on their physical and mental well-being. The results underscore the need for interventions that contribute to the well-being of minors in transit in the LAC and sustainable, safe, long-term solutions for individuals and families who are forced to migrate from their countries of origin to avoid worse consequences on their health.

**Data sharing statement**: The data underlying this article will be shared on reasonable request to the corresponding author.

## Funding sources

This research did not receive any specific grant from funding agencies in the public, commercial, or non-profit sectors.

## CRediT authorship contribution statement

**Zeus Aranda:** Writing – review & editing, Writing – original draft, Visualization, Validation, Software, Resources, Project administration, Methodology, Investigation, Formal analysis, Data curation, Conceptualization. **Ana Cristina Sedas:** Writing – review & editing, Validation, Methodology, Conceptualization. **Daniel Bernal:** Writing – review & editing, Validation, Methodology, Conceptualization. **José Pulido-Manzanero:** Writing – review & editing, Visualization, Methodology, Conceptualization. **Enrique Regidor:** Writing – review & editing, Visualization, Methodology, Conceptualization. **Anna M. Mandalakas:** Writing – review & editing, Visualization, Validation, Resources, Methodology, Conceptualization. **Karla Fredricks:** Writing – review & editing, Writing – original draft, Visualization, Validation, Resources, Methodology, Conceptualization.

## Declaration of competing interest

The authors declare that they have no known competing financial interests or personal relationships that could have appeared to influence the work reported in this paper.

## Appendix Supplementary materials

Image, application 1
