# Supplementary material for: Health status and living conditions of north-bound migrant minors in Mexico at place of origin and during the migration journey: A cross-sectional, mixed-methods study
Source: J Migr Health. 2025 Dec 30;13:100390. doi: 10.1016/j.jmh.2025.100390 (PMC12834919; doi:10.1016/j.jmh.2025.100390)
Supplement: Supplementary file 1 [file mmc1.docx]

**SUPPLEMENTARY MATERIAL**

Table of contents

1. Supplementary information on the tools used for data collection (page 2-3)
2. Supplementary table 1. Guide for interpretation of the lowest and highest values of the subscales of the 28 Item Short Child Health Questionnaire Parent Form and the 47 Item Short Infant and Toddler Quality of Life Questionnaire Parent Form included in the study (pages 4-5)
3. Supplementary table 2. Detailed results for The Accountable Health Communities Health-Related Social Needs Screening Tool (pages 6-7)
4. Supplementary table 3. Diagnosed health conditions (page 8)
5. Supplementary figure 1. Countries through which the minors travelled in their migratory journey (n=200) (page 9)
6. Supplementary figure 2. Injuries of the minors during the journey (n=200) (page 10)
7. Supplementary figure 3. Health discomforts of the minors during the journey (n=200) (page 11)

**Supplementary information on the tools used for data collection**

To assess the health status of minors, the short Spanish versions of the Child Health Questionnaire (CHQ) for children and adolescents aged 5 to 17 years and the Infant Toddler Quality of Life Questionnaire (ITQOL) for children aged 12 months or older under 5 years of age were used.^1^ Both tools have been widely used and validated in different contexts and populations.^2^ The Spanish version of the CHQ has been adapted and validated for the Mexican population;^3^ in addition, it has been used with caregivers of migrant children of Mexican, Guatemalan, Honduran and Salvadoran origin who had recently arrived in the United States (US) in a previous study, obtaining a high reliability for that sample.^4^

To characterize the health-related social needs of the population of minors in their country of origin, the "Accountable Health Communities Health-Related Social Needs Screening Tool" designed by the Accountable Health Communities program funded by the US Centers for Medicare and Medicaid Services (CMS) was used.^5^ The tool has been widely used with beneficiaries of the US federal health insurance programs, Medicaid and Medicare.^6^ Although there are other tools for assessing health-related social needs,^7–10^ the tool designed by CMS has several characteristics that made it the best choice for our study. In its design, those social dimensions for which there is ample evidence available of the association between this and health status were considered central, these being housing, housing supplies, food, transport, and safety. In addition, the design of the questions was sufficiently broad, simplified and easy to understand and to be based on validated tools.^11^ Although the tool in its entirety has not been implemented and validated outside the US, some of the tools used in its design have been (such as the Hurt, Insult, Threaten, and Scream [HITS] instrument or the Hunger Vital SignTM).^11^ The questionnaire, available and validated in Spanish,^12^ has adaptations to be answered by a caregiver, as in the case at hand.^13^ In addition, a high implementability of the tool with caregivers of Spanish-speaking pediatric patients has been reported.^14^

References:

1 QualityMetric. Pediatric PRO Health Surveys. 2024. https://www.qualitymetric.com/pediatric-pro-health-surveys/ (accessed Feb 26, 2024).

2 QualityMetric. CHQ bibliography. 2023 https://www.qualitymetric.com/wp-content/uploads/2023/12/QM_Bibliography.pdf (accessed Feb 26, 2024).

3 Duarte C, Ruperto N, Goycochea M, *et al.* The Mexican version of the Childhood Health Assessment Questionnaire (CHAQ) and the Child Health Questionnaire (CHQ). *Clin Exp Rheumatol* 2001; **19**: S106-10.

4 Mercado A, Venta A, Henderson C, Pimentel N. Trauma and cultural values in the health of recently immigrated families. *J Health Psychol* 2021; **26**: 728–40.

5 Centers for Medicare & Medicaid Services. The Accountable Health Communities Health-Related Social Needs Screening Tool. 2023 https://www.cms.gov/priorities/innovation/files/worksheets/ahcm-screeningtool.pdf (accessed Feb 26, 2024).

6 Bosold A, Abrams Weintraub T, Cowen K, Talwar-Hebert M, Abowd Johnson K, Barolín N. Health-Related Social Needs Screening: Promising Practices From the Accountable Health Communities Model. *Health Promot Pract* 2023. DOI:10.1177/15248399231213582.

7 Moen M, Storr C, German D, Friedmann E, Johantgen M. A Review of Tools to Screen for Social Determinants of Health in the United States: A Practice Brief. *Popul Health Manag* 2020; **23**: 422.

8 Sokol R, Austin A, Chandler C, *et al.* Screening children for social determinants of health: A systematic review. *Pediatrics* 2019; **144**. DOI:10.1542/PEDS.2019-1622/38457.

9 Henrikson NB, Blasi PR, Dorsey CN, *et al.* Psychometric and Pragmatic Properties of Social Risk Screening Tools: A Systematic Review. *Am J Prev Med* 2019; **57**: S13–24.

10 Narayan A, Raphael J, Rattler T, Bocchini C. Social determinants of health: Screening in the clinical setting. Houston, 2018 https://www.texaschildrens.org/sites/default/files/uploads/documents/83176 BRIEF Social Determinants of Health Policy Digital.pdf (accessed Feb 26, 2024).

11 Billioux A, Verlander K, Anthony S, Alley D. Standardized Screening for Health-Related Social Needs in Clinical Settings: The Accountable Health Communities Screening Tool. *NAM Perspectives* 2017; **7**. DOI:10.31478/201705B.

12 Lewis C, Wellman R, Jones SW, *et al.* Comparing the performance of two social risk screening tools in a vulnerable subpopulation. *J Family Med Prim Care* 2020; **9**: 5026.

13 Centers for Medicare & Medicaid Services. A Guide to Using the Accountable Health Communities Health-Related Social Needs Screening Tool: Promising Practices and Key Insights. 2023 https://www.cms.gov/priorities/innovation/media/document/ahcm-screeningtool-companion (accessed Feb 26, 2024).

14 Gray TW, Podewils LJ, Rasulo RM, Weiss RP, Tomcho MM. Examining the Implementation of Health-Related Social Need (HRSN) Screenings at a Pediatric Community Health Center. *J Prim Care Community Health* 2023; **14**. DOI:10.1177/21501319231171519.

**Supplementary table 1. Guide for interpretation of the lowest and highest values of the subscales of the 28 Item Short Child Health Questionnaire Parent Form and the 47 Item Short Infant and Toddler Quality of Life Questionnaire Parent Form included in the study. Adapted from QualityMetric, 2024.**

| **47 Item Short Infant and Toddler Quality of Life Questionnaire Parent Form (ITQOL-SF47)** | **Sub-scale** | **Lowest possible value** | **Highest possible value** |
| --- | --- | --- | --- |
|  | Overall Health Scale | The child's health in general is rated as "poor". | The child's health in general is rated as "excellent". |
|  | Physical Abilities Scale | Child is considerably limited in performing physical activities such as eating, sleeping, grasping, and playing due to health problems. | Child performs all types of physical activities such as eating, sleeping, grasping, and playing without limitations due to health problems. |
|  | Growth and Development Scale | Parent is very dissatisfied with development (physical growth, motor, language, cognitive), habits (eating, feeding, sleeping) and overall temperament. | Parent is very satisfied with development (physical growth, motor, language, cognitive), habits (eating, feeding, sleeping) and overall temperament. |
|  | Bodily Pain/Discomfort Scale | Child has extremely, frequent and limiting bodily pain/discomfort. | Child has no pain or limitations due to pain/discomfort. |
|  | Temperament and Moods Scale | Child very often has certain moods and temperaments, such as sleeping/eating difficulties, crankiness, fussiness, unresponsiveness and lack of playfulness and alertness. | Child never has certain moods and temperaments, such as sleeping/eating difficulties, crankiness, fussiness, unresponsiveness and lack of playfulness and alertness. |
|  | Global Behavior Scale | The child's behavior in general is rated as "poor". | The child's behavior in general is rated as "excellent". |
|  | Combined Behavior Scale | Child very often exhibits behavioral problems, such as not following directions, hitting, biting others, throwing tantrums, and being easily distracted, while positive behavior, such as ability to cooperate, to appear sorry, and to adjust to new situations is seldom shown. | Child never exhibits behavioral problems, such as not following directions, hitting, biting others, throwing tantrums, and being easily distracted, while positive behavior, such as ability to cooperate, to appear sorry, and to adjust to new situations is frequently shown. |
|  | General Health Perception Scale | Parent believes child's health is poor and likely to get worse. | Parent believes child's health is excellent and will continue to be so. |
|  | Parental Impact - Emotional Scale | Parent experiences a great deal of emotional worry/concern as a result of child's physical and/or psychosocial health and/or growth and development. | Parent does not experience feelings of emotional worry/concern due to child's physical and/or psychosocial health and/or growth and development. |
|  | Parental Impact - Time Scale | Parent experiences a lot of limitations in time available for personal needs due to child's physical and/or psychosocial health and/or growth and development. | Parent does not experience limitations in time available for personal needs due to child's physical and/or psychosocial health and/or growth and development. |
|  | Family Cohesion Scale | Family's ability to get along is rated as "poor". | Family's ability to get along is rated as "excellent". |
| **28 Item Short Child Health Questionnaire Parent Form (CHQ-PF28)** | Global Health Scale | The child's health in general is rated as "poor". | The child's health in general is rated as "excellent". |
|  | Physical Functioning Scale | Child is limited a lot in performing all physical activities, including self-care, due to health. | Child performs all types of physical activities, including the most vigorous, without limitations due to health. |
|  | Role/Social Limitations - Emotional Behavior Scale | Child is limited a lot in schoolwork or activities with friends as a result of behavior/emotional problems. | Child has no limitations in schoolwork or activities with friends due to behavior/emotional problems. |
|  | Role/Social Limitations - Physical Scale | Child is limited a lot in schoolwork or activities with friends as a result of physical health. | Child has no limitations in schoolwork or activities with friends as a result of physical health. |
|  | Bodily Pain/Discomfort Scale | Child has extremely severe, frequent, and limiting bodily pain/discomfort. | Child has no pain or limitations due to pain/discomfort. |
|  | Behavior Scale | Child very often exhibits aggressive, immature, or delinquent behavior. | Child never exhibits aggressive, immature, or delinquent behavior. |
|  | Global Behavior Scale | The child's behavior in general is rated as "poor". | The child's behavior in general is rated as "excellent". |
|  | Mental Health Scale | Child has feeling of anxiety and depression all of the time. | Child feels peaceful, happy, and calm of all the time. |
|  | Self Esteem Scale | Child is very dissatisfied with abilities, looks, family/peer relationships and life overall. | Child is very satisfied with abilities, looks, family/peer relationships and life overall. |
|  | General Health Perception Scale | Parent believes child's health is poor and likely to get worse. | Parent believes child's health is excellent and will continue to be so. |
|  | Parental Impact - Emotional Scale | Parent experiences a great deal of emotional worry/concern as a result of child's physical or psychological health. | Parent does not experience feelings of emotional worry/concern due to child's physical or psychological health. |
|  | Parental Impact - Time Scale | Parent experiences a lot of limitations in tie for personal needs due to child’s physical or psychological health. | Parent does not experience limitations in time for personal needs due to child's physical or psychological health. |
|  | Family Activities Scale | The child's health very often limits and interrupts family activities or is a source of family tension. | The child's health never limits or interrupts family activities nor is a source of family tension. |
|  | Family Cohesion Scale | Family's ability to get along (relationships) is (are) rated "poor". | Family's ability to get along (relationships) is (are) rated "excellent". |
|  | Physical Component Summary Measure (summary of different items related to the child's physical health) | Norm-based scoring (NBS) is used to calculate the two summary scores. NBS standardizes component scores using the means and standard deviations from a U.S. general population normative sample. The aggregated scores are standardized using a linear t-score transformation (mean of 50 and a standard deviation of 10). Summary scores below 50 can be interpreted as being below the U.S. general population norm (average), while scores above 50 can be interpreted as above the U.S. general population norm. | |
|  | Mental Component Summary Measure (summary of different items related to the child's mental health) |  |  |

**Supplementary table 2. Detailed results for The Accountable Health Communities Health-Related Social Needs Screening Tool**

|  | **n (%)** |
| --- | --- |
| **Housing situation** | n=196 |
| Did not have a stable place to live | 57 (29.08) |
| Had a place to live but worried about losing it | 15 (7.65) |
| Had a stable place to live | 124 (63.27) |
| **Problems in the place where you lived (multiple choice)** |  |
| Lack of smoke detectors or smoke detectors not working | 194 (98.98) |
| Mold | 91 (46.43) |
| Water leaks | 63 (32.14) |
| Pests such as insects, ants or rats | 59 (30.1) |
| Oven or stove that did not work | 14 (7.14) |
| Lack of heat | 10 (5.1) |
| Lead paint or plumbing | 0 |
| **Concern that your food would run out before you had money to buy more (year before departure)** |  |
| Never | 46 (23.47) |
| Occasionally | 19 (9.69) |
| Frequently | 131 (66.84) |
| **The food you bought was not enough or you did not have money to buy more (year before departure)** |  |
| Never | 46 (23.47) |
| Sometimes | 19 (9.69) |
| Frequently | 131 (66.84) |
| **Lack of transportation prevented you from going to medical appointments, meetings, work, or getting things you needed for daily life (year before departure)** |  |
| No | 111 (56.63) |
| Yes | 85 (43.37) |
| **Electricity, gas, fuel, or water company threatened to shut off services to your home (year prior to departure)** |  |
| They canceled it | 5 (2.55) |
| Yes | 15 (7.65) |
| No | 176 (89.8) |
| **Someone physically hurt the child** |  |
| Never | 116 (59.18) |
| Rarely | 2 (1.02) |
| Sometimes | 19 (9.69) |
| Quite often | 5 (2.55) |
| Frequently | 54 (27.55) |
| **Someone insulted the child or talked down to him or her** |  |
| Never | 115 (58.67) |
| Rarely | 2 (1.02) |
| Sometimes | 20 (10.2) |
| Quite often | 5 (2.55) |
| Frequently | 54 (27.55) |
| **Someone threatened the child with harm** |  |
| Never | 114 (58.16) |
| Rarely | 2 (1.02) |
| Sometimes | 21 (10.71) |
| Quite often | 5 (2.55) |
| Often | 54 (27.55) |
| **Someone yelled at the child or cursed at him/her** |  |
| Never | 115 (58.67) |
| Rarely | 2 (1.02) |
| Sometimes | 20 (10.2) |
| Quite often | 5 (2.55) |
| Often | 54 (27.55) |

**Supplementary table 3. Diagnosed health conditions**

|  | **n (%)** |
| --- | --- |
|  | n=200 |
| **Origin** |  |
| **Cases for each disability (multiple choice)** |  |
| Neurodevelopmental Disorder | 3 (1.5) |
| Visual impairment | 2 (1) |
| Intellectual sequelae due to trauma | 1 (0.5) |
| **Cases for each non-infectious disease (multiple choice)** |  |
| Asthma | 17 (8.5) |
| Anemia | 2 (1) |
| Atopic dermatitis | 2 (1) |
| Epilepsy | 2 (1) |
| Allergic sinusitis | 2 (1) |
| Innocent murmur | 2 (1) |
| Chronic tonsillitis | 1 (0.5) |
| Infantile arthritis | 1 (0.5) |
| Epistaxis and plateletopenia | 1 (0.5) |
| Pulmonary hypoplasia | 1 (0.5) |
| Elevated blood lead level | 1 (0.5) |
| Lacrimal duct obstruction | 1 (0.5) |
| Pre-diabetes | 1 (0.5) |
| **Cases by infectious disease (multiple choice)** |  |
| Bacterial stomach infection | 3 (1.5) |
| Lower respiratory tract infection | 2 (1) |
| **Journey** |  |
| **Non-infectious disease cases (multiple choice)** |  |
| Anemia | 3 (1.5) |
| Jaundice | 1 (0.5) |
| **Infectious disease cases (multiple choice)** |  |
| Middle ear infection | 4 (2) |
| Intestinal parasitic infection | 2 (1) |
| Fungal skin infection | 2 (1) |
| Lower respiratory tract infection | 2 (1) |
| Malaria | 2 (1) |
| Chickenpox | 2 (1) |
| Acute tonsillitis | 1 (0.5) |
| Scabies | 1 (0.5) |
| Bacterial stomach infection | 1 (0.5) |
| Urinary tract infection | 1 (0.5) |
| Leishmaniasis | 1 (0.5) |
| Peritonitis | 1 (0.5) |

**Supplementary figure 1. Countries through which the minors travelled in their migratory journey (n=200)**


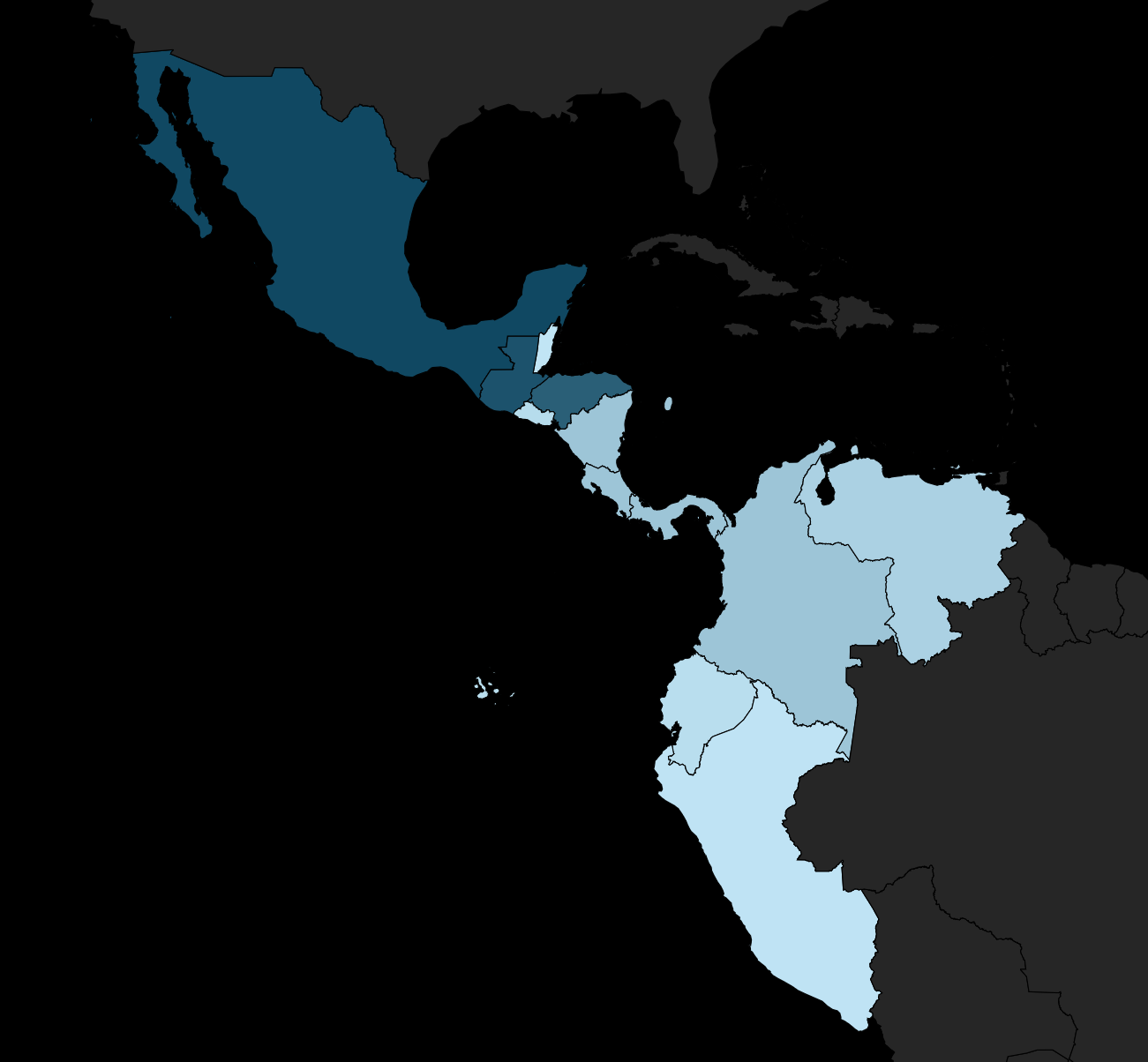

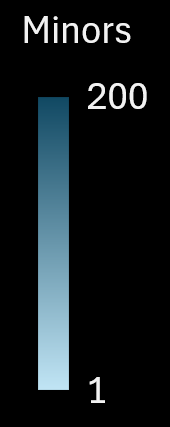


**Supplementary figure 2. Injuries of the minors during the journey (n=200)**

**Supplementary figure 3. Health discomforts of the minors during the journey (n=200)**
